# Consolidating RRI and Open Science: understanding the potential for transformative change

**DOI:** 10.1186/s40504-020-00103-5

**Published:** 2020-09-01

**Authors:** Clare Shelley-Egan, Mads Dahl Gjefsen, Rune Nydal

**Affiliations:** 1 Work Research Institute, OsloMet – Oslo Metropolitan University, Postboks 4 St. Olavs plass, 0130 Oslo, Norway; 2 Programme for applied ethics, Department of Philosophy and Religious Studies, NTNU – Norwegian University of Science and Technology, NO-7491 Trondheim, Norway

**Keywords:** Europe, Open Science, Research and innovation policy, Responsible Research and Innovation, Transformative change

## Abstract

In European research and innovation policy, Responsible Research and Innovation (RRI) and Open Science (OS) encompass two co-existing sets of ambitions concerning systemic change in the practice of research and innovation. This paper is an exploratory attempt to uncover synergies and differences between RRI and OS, by interrogating what motivates their respective transformative agendas. We offer two storylines that account for the specific contexts and dynamics from which RRI and OS have emerged, which in turn offer entrance points to further unpacking what ‘opening up’ to society means with respect to the transformative change agendas that are implicit in the two agendas. We compare differences regarding the ‘how’ of opening up in light of the ‘why’ to explore common areas of emphasis in both OS and RRI. We argue that while both agendas align with mission-oriented narratives around grand societal challenges, OS tends to emphasize efficiency and technical optimisation over RRI’s emphasis on normative concerns and democracy deficits, and that the two agendas thus contrast in their relative legitimate emphasis on *doable* outcomes versus *desirable* outcomes. In our conclusion, we reflect on the future outlook for RRI and OS’ co-existence and uptake, and on what their respective ambitions for transformation might mean for science-society scholars and scholarship.

## Introduction

In European research and innovation policy, Responsible Research and Innovation (RRI) and Open Science (OS) encompass two co-existing sets of ambitions concerning systemic change in the practice of research and innovation. This paper is an exploratory attempt to uncover synergies and differences between RRI and OS. Such a comparison is important because RRI and OS both offer prescriptions and specific routes regarding *how* existing research and innovation systems *could* o*r should* be transformed to better ends. Both ambitions prescribe transformative change of the research and innovation (R&I) system. Both, we argue, could be seen to prescribe ways of “opening up” the R&I system to society – but in ways that may or may not support the same ends. At first glance, RRI and OS may appear to align well. If RRI aims to facilitate solutions to the grand challenges faced by society by bringing a range of societal actors together in an interactive, transparent and responsive process, OS could be understood as calling attention to the need for science to adapt to the new era of information technology in order to enable collaboration across disciplines and sectors needed to solve grand challenges. This paper aims to explore whether we should understand the two concepts as mutually supportive of the same ends or not. As scholars engaged in both RRI and OS projects, this has become a pressing issue for us. What does the co-existence of RRI and OS initiatives imply for those of us who study, offer advice on and aim to be an integral part of science-society dynamics? Should we understand RRI and OS as successive phases in a process of transition?

In order to clarify whether RRI and OS support the same ends we take as our point of departure that both take different forms (research topics, policy frameworks, visions), with respective differences and emphases regarding their precise meaning or rationale, but that the two nonetheless can be meaningfully compared as transformative change agendas pertaining to research and innovation. Our concern therefore is less with different programmatic definitions, and more with the logics and ambitions of the prescribed research practices.

We argue that we need to understand what motivates a transformative agenda, i.e. why do we *need* to open up the R&I system? To what future ends are the prescribed changes in the contemporary R&I system directed? In order to explore this question, we begin in section two by offering two storylines that account for the specific contexts and dynamics from which RRI and OS have emerged, respectively. These storylines clarify our understanding of RRI and OS supporting our analysis of what ‘opening up’ to society means with respect to the transformative change agendas that are implicit in RRI and OS. In section three, we move on to compare differences regarding the ‘how’ of opening up in light of the ‘why’ to explore two areas of emphasis in both OS and RRI, namely engagement with publics and stakeholders, on the one hand, and mission-focused interdisciplinarity, on the other. We argue that while both agendas express mission-oriented narratives around grand societal challenges (Mazzucato 2018), OS tends to emphasise efficiency and technical optimisation over RRI’s emphasis on normative concerns and democracy deficits. We argue that the two agendas thus contrast in their relative legitimate emphasis on *doable* outcomes versus *desirable* outcomes. In our conclusion, we reflect on the future outlook for RRI and OS’ co-existence and uptake, and on what their respective ambitions for transformation might mean for science-society scholars and scholarship.

## Storylines of two movements, RRI & OS

### RRI

As a policy concept, Responsible Research and Innovation emerged in 2011, in the course of discussions around socio-technical integration with respect to the European Commission’s Science in Society programme (Owen et al. 2012). However, as noted by many scholars and commentators, RRI is a concept with a storied pre-history (Felt 2018, Rip 2014). The RRI concept has a strong academic foundation, with its roots in various traditions that seek to enhance the integration of science and society, such as technology assessment, anticipatory governance and Ethical, Legal and Societal Aspects (ELSA) (Zwart et al. 2014, Felt, 2018). In particular, the ELSA programme was an important precedent to RRI (Zwart et al. 2014, Felt, 2018). In the EU policy context, ELSA was introduced in the Fourth Framework Programme (1994–98) as a label for developing and supporting research ELSA research concerning new and emerging science and technologies. Funding was mobilised to support ELSA research into large scale publicly funded research programmes within post-genomics (Nydal et.al 2015). Another important precedent for RRI was the notion of “responsible development” which emerged – again, primarily in the European Union - as a science and innovation policy response to concerns about nanotechnologies (Rip 2014, Grunwald, 2014, Shelley-Egan et al. 2018). Concerns about the potential negative impacts of nanotechnology on human health and the environment (NFR 2005, NSF 2001, Royal Society and Royal Academy of Engineering 2004) and the felt need to get the development of nanotechnology “right from the very beginning” led to the coupling of nanotechnologies and responsibility.

RRI became an important innovation policy issue for a variety of R&I actors for a myriad reasons including the need to orient new technologies toward societal challenges, to prevent catastrophic disasters such as those associated with asbestos, chlorofluorocarbons (CFCs) and so on, and to establish trust and confidence of the public and other stakeholders in safe and effective processes, products and systems of innovation (Sutcliffe 2011). RRI became linked to other emerging technologies, ranging from synthetic biology and geoengineering to robotics and ICT (Guston et al. 2014; Jirotka et al. 2017).

RRI has emerged not just as an approach to identifying, analysing and responding to individual instances of ethical or societal controversies, but to building systematic anticipation and inclusive co-development into the research (planning) process itself. RRI, we claim, should primarily be seen as a movement calling attention to normative aspects of the research and innovation system. Furthermore, the RRI term, in the scholarly circles that promote it, expresses a key *normative diagnosis*, namely that the established professional identities and training regimes of the traditional social contract between science and society are part of the problem. There is a demarcation rationality of scientists in force (Glerup and Horst 2014) – a rationality that appraises science as having a high level of autonomy from other actors, along with an exemption from taking responsibility for decisions on the direction of science in and for society. RRI challenges the demarcation rationality of the social contract. Crucially in our view, RRI calls for a re-thinking of the social contract, that is a rethinking of the division of moral labour between science and society (Rip and Shelley-Egan 2010, Rip 2014, Myskja et.al 2014, Rip, 2016). Such roles are sustained by explicit and implicit shared professional norms expressed and maintained in well-established practices, training programmes and institutional arrangements. RRI seeks to extend and reconfigure established roles and responsibilities so as to include future societal impacts of technological development (Rip and Shelley-Egan 2010, von Schomberg 2013, Fisher and Rip 2013).

The strength of the RRI concept, as articulated by Felt (2018), “( …) cannot be seen in its radical newness, but rather in its capacity to re-articulate long-standing claims about innovation and society in new ways” (Felt. 2018: 7). This implies “( …) creating spaces for fostering more collective forms of engagement; experimenting in new constellations of actors; and reconsidering the diverse values at stake in innovation” (ibid. 7). Academic definitions of RRI share various threads, with an emphasis on the dimensions of anticipation, inclusion, reflexivity and responsiveness (Stilgoe et al. 2013). This can be further articulated as a grand vision for “( …) taking care of the future through collective stewardship of science and innovation in the present” (ibid: 1570).

Critically, this endeavour should proceed with the upstream involvement and engagement of societal actors – researchers, innovators, civil society actors and citizens – working together to co-create societally desirable and responsive research and innovation. In order to draw attention to the challenge at hand, RRI researchers typically call for *experiments.* Such experiments aim to open up existing practices, and experiment with new forms of collective engagement, with the aim of engaging and aligning the interests of all stakeholders. The ends of technical processes informed by societal engagement cannot be predefined on scientists’ terms alone – thus, societal engagement also involves a certain level of indeterminacy with respect to the output and goals of genuine co-creation. It is to these normative ends that the experiments are to be ultimately evaluated, at least in theory.

Interdisciplinarity is another key facet of RRI, with the promotion of collaboration between the social sciences and humanities (SSH) and the natural sciences and engineering in order to advance the “science with and for society” ambition (Delgado and Åm 2018). This reflects a call for a collaborative mode of work rather than a cooperative mode. The standard cooperative mode assumes a clear division of labour underpinned by the demarcation rationality mentioned above. Conversely, a “collaborative mode proceeds from an interdependent division of labour on shared problems” (Rabinow and Bennett 2009: 266).

At EU level, RRI is a cross-cutting strategy across the Horizon 2020 funding programme – due to wrap up at the end of 2020 - and is positioned as having a key role to play in addressing grand societal challenges. The EC’s framing of RRI is underpinned by five policy agendas, namely, open access, ethics, science education, public engagement and gender, on which action should be taken (European Commission, undated). As noted by Rip (2014,3), these RRI policy keys “have more to do with the bureaucracy of maintaining SwafS/RRI as a cross-cutting theme than with the conceptual foundations of RRI”, mentioned previously. These policy agendas tend to sit somewhat uneasily with the more academic understandings of RRI – where the concern is that RRI activities may be re-inscribed into the dynamics of existing normative regimes (Owen and Pansera 2019).^1^

### Open Science

Open Science is gaining increasing prominence at national and supranational levels, as evidenced, for example, in the European Commission’s (EC) ‘3Os’ or the three strategic research priorities underlying current EU research and innovation policy, namely *Open Innovation, Open Science and Open to the World* (European Commission, 2016). Open science intuitively resonates with long-established academic ideals, such as Mertonian principles of communism and universalism (Merton 1973). Moreover, science’s capacity for self-correction, collective improvement and objective to serve the public good all derive from a culture of openness. Indeed, “Open inquiry is at the heart of the scientific enterprise” (The Royal Society 2012: 7).

This tradition in science is an important aspect of the mobilising strength of the open science movement. Open Science, however, calls for a further ‘opening up’ of the research process by extending the principle of openness to *all* aspects of the research process (Nielsen, 2012, e-IRG 2016). One key aspect of the Open Science movement rests on an appeal to other scientists: If we are to stay true to our own ideals, we should ‘open up’ for two connected reasons, maintaining quality on the one hand, and enhancing scientific capacity to serve the public good on the other. Our concern in this paper is primarily with the latter but for context we briefly give an account of each reason here.

First, historical scientific norms of openness and transparency are increasingly difficult to maintain, calling for new forms of *intelligent* openness. 1.3–1.5 million peer reviewed papers are published every year in what has now exceeded 24,000 scientific journals (plus an additional increasing number of journals that do not meet minimum scientific standards) (Jinha 2010, Schatz 2014). Publication bottlenecks put increased pressure on the peer review system (Noorden 2013), and lead to a ‘data deluge’ in both natural and social sciences (Hey et al. 2009, Bell et al. 2009; Elmqvist and Pourang 2013). We are thus in a situation in which strategies for data collection and analysis are changing, challenging traditional notions of transparency in the process (Tenenbaum et al., 2014, Baker 2016). These developments can be seen as a ‘closing’ of the scientific cultures in the sense that it has become difficult to access, effectively communicate, assess and exploit research findings. The very foundation of what ensures the quality of scientific inquiry is at stake, a concern that for instance has been discussed under the heading of the ‘reproducibility crisis’. We are at a point in history, as stated in a preface to a National Science Foundation report on reproducibility, in which “it is necessary to shore up and reaffirm the foundations of scientific inquiry. There is a new sense of urgency in maintaining rigorous procedures for verification and validation of scientific knowledge” (NSF 2017:7). We have, in other words, reached a situation where we cannot simply use the term ‘science’ assuming openness lies at the heart of the enterprise but need to speak of ‘open science’ (Watson 2015). The “changes that are needed go to the heart of the scientific enterprise and are *much more than a requirement to publish or disclose more data.* [It] requires *[.. a] more intelligent openness*” (The Royal Society 2012:7). We need to find ways to ‘open up science’ that in fact calls for a new form of doing science – enabled by computational networks. Intelligent openness refers to practical issues of accessibility, intelligibility, and scrutinisability to enable judgements to be made about the reliability and competence of those who make scientific claims. The first reason for opening science then, implies a radical opening of all aspects of research to maintain quality. This is expressed in separate transformative initiatives like open -data, −access, −methodology, −source, −peer review, and -education (Watson 2015).

The second reason for opening up has not received enough attention. Measures to support ongoing OS initiatives are needed not only to reaffirm the foundations of science, but also to fully exploit novel scientific opportunities of the collaborative computer enabled networks that are coming into place. Over the last two decades, digitalisation has already made a strong impact on the manner in which research is performed across all phases of the research life cycle, from discovery, planning, funding, project implementation, analysis, publication and outreach to the evaluation of research (Science Europe 2017). These networks need to be continuously moulded to fully exploit the expected potential of digitalisation.

The potential of big data is often highlighted with respect to the OS vision for transformation of the R&I system. Large scale production and sharing of data (including experimental data) have stimulated novel forms of analytics with greater emphasis on data mining and big data analytics. These developments have inspired the formulation of a vision of science referred to as the ‘fourth paradigm of scientific research” in which the speed of development of any research field is predicted to depend on how well the field manages to adopt digital technologies to maintain and enhance collaboration. We are now in a new digital paradigm in which the “capacity to measure, store, analyse, and visualize data is the new reality to which science must adapt” (Hey et al., 2009: 210).^2^

The second reason for opening science then, manifests a vision of digitalisation that in turn require adaptations to the new era of information technology. As such, OS is in the making, it is at the same time a vision and a series of explorative practices expressing the vision. A EU consultation presents a list of terms being used interchangeably including ‘participatory science’, ‘better science’, ‘science highway’, ‘open research’, ‘open scholarship’, ‘open science’ and ‘Science 2.0’ (European Commission, 2015b:6). The term Science 2.0 is particularly illustrative, along with alternative terms such as Cyberscience 2.0 and eScience (Bartling and Friesike 2014). Science is imagined in computational terms. Science as we know it is becoming outdated and in need of an upgrade, or even a ‘reboot’ - and efforts are needed to make it happen (Dijstelbloem et al. 2013, Katarzyna and Osimo 2016).

The inclusion of ‘participatory science’ and ‘open scholarship’ in the abovementioned list suggests that an upgrade of science may also need to include an ‘opening up’ of science to society. The digitalisation of science requires collaborative efforts across academic disciplines and societal actors and practices. Importantly, this transformation of the way in which research is carried out, according to the 3Os strategy document (European Commission, 2016), is to support an upgrade of innovation. Open innovation 2.O aims at “building and funding ecosystems for co-creation” and working “towards a networked, multi-collaborative innovation ecosystem”, in which innovation is not seen as an “isolated activity without considering the consequences for its entire economic and social environment” (European Commision, 2014 4–5). The 3Os strategy articulates a grand vision in which research institutions align themselves with other stakeholders in the existing R&I system to support innovation; “The EC, Member States, universities and public research organizations, corporate sector financial institutions, local communities and their citizens have no option but to advocate and to support an open, networked and collaborative innovation-led growth on which, in different ways, their own intellectual, operational and financial vitality will increasingly depend” (European Commision, 2014: 3).

Open Science, like RRI, calls for transformative changes of science although with a different point of departure, namely that science needs to ‘open up’ to maintain its own Mertonian ideals. Collaborative coordinated efforts within and outside academia are needed not only to maintain capacity for self-correction in current research systems, but also to develop new platforms or highways for research to ensure science capacity to meet societal needs. Thus, what Open Science fundamentally offers are *opportunities*, not only to a broad research community, but also to society at large.

## Comparing prescriptive actions for transformation

RRI and OS have emerged out of different contexts, are underpinned by different drivers and reflect certain trends in the continuously evolving R&I system. In our account, RRI has emerged in the context of scholarly work in the social sciences and humanities concerned with normative deficiencies of existing R&I systems. OS has emerged in research communities concerned with epistemic deficiencies in existing R&I systems.

On the face of it, RRI and OS’s transformative agendas broadly align in key areas, as seen, for instance, in the emphasis on research integrity in OS. Open Science can for instance be seen to open up new opportunities to support and strengthen a culture of research integrity by diffusing knowledge at an earlier stage in the research process, opening up access to data and research results, facilitating reproducible research, and reducing and discouraging cases of fabrication, falsification, plagiarism and other unacceptable research practices (LERU 2018). The cultivation of “system integrity” in this manner is viewed by some as the most efficient means of deterring and detecting poor or fraudulent practices (The Royal Society 2012). OS can also be viewed as a mechanism with which to bring together a network of stakeholders to address uniquely complex questions that could not be addressed by any one individual or nation state. This rationale echoes RRI’s emphasis on dialogue across societal groups and sectors to define priorities and concerns, thereby more effectively marshalling research and innovation to address “wicked” problems (Rittel and Webber 1974).

At an overall level then, RRI and OS may align well. Both movements express transformative agendas that may be said to align towards the same aim: to address the grand challenges of our time. Here, we should note that the relevance for grand challenges is far more explicit in the RRI-focused discourse than in the OS discourse, and that the OS relevance and alignment with grand challenges may be viewed as less direct than in RRI, and as consisting of the assumed societal value brought about by OS’s more general efforts to improve capacities in scientific activity. For RRI, the alignment with grand challenges is far more integral to its core logic and claims to relevance, where related problem diagnoses justifying RRI approaches are concerned with issues of interconnectedness, systemic failures, indeterminacy, wickedness and complexity. These issues are viewed as being at the heart of many of societal grand challenges’ evasion of mere technical, expert, and rationalistic solutions. Thus societal grand challenges are in need of the types of anticipatory and reflexive activities generally advocated in RRI. The prescriptions of RRI and OS towards grand challenges can be considered similarly as well; both RRI and OS call attention to ways of “opening up” the R&I system to society. If RRI aims to facilitate solutions to the grand challenges faced by society by bringing a range of societal actors together in an interactive, transparent and responsive process, OS could be understood as calling attention to the need for science to adapt to the new era of information technology in order to enable collaboration across disciplines and sectors needed to solve the grand challenges.

Differences between the two agendas become apparent when one looks at how ambitions for ‘opening up’ are translated into prescriptions for new measures and practices. For example, when taken up and supported by policy actors, mechanisms for RRI have tended to emphasise new practices for continuous and inclusive input to research and development trajectories, while mechanisms around OS emphasise new efforts around data harmonisation as well as data-sharing infrastructures. Considerable resources are needed to realise Open Science ambitions, as evidenced by examples such as the European Open Science Cloud project, whereby the EC initiated funding for part of a € 6.7 billion virtual repository to store and provide access to vast amounts of publicly funded research data. The project presented considerable logistical and coordination challenges around data harmonisation and the need for new browsing and annotation tools, and the EC’s co-funding was intended to entice member states and private sources to co-fund the effort in pursuit of new benefits to research and private industry (Nature 2017). By contrast, RRI efforts to ‘open up’ tend to be project-based, with limited coordination across and between initiatives. Nevertheless, studies are increasingly showing that RRI does make a difference (Mejlgaard et al. 2018a, Stilgoe 2018). Indeed, EU-funded projects in particular have brought together “important elements of opening up science and innovation to societal actors through an institutionalized concept and the promotion of concrete structural changes” (Mazzonetto and Simone 2018: 2). Work on RRI – both conceptual and practical – implemented through the European Commission’s Framework Programmes (FP) has delivered a plethora of methods, theories and practices of RRI (Braun and Griessler 2018). As noted by Jack Stilgoe in a policy brief for the MORRI (Monitoring the evolution and benefits of Responsible Research and Innovation in Europe) project, aspirational agendas such as the RRI agenda now need to be coupled with measurable indicators (Stilgoe 2018: 2). To that end, the MORRI project offers indicators *of* (e.g. number of publications that are open access) and *for* RRI (e.g. data on the views of scientists). The MORRI project observed a socialising effect of participating in FP research projects on individual researchers, while a positive trend was demonstrated with respect to the treatment of RRI keys in academic organisations. In addition, European research and coordination activities have “opened up avenues for learning about RRI between countries and have built new professional communities of researchers, administrators, policy-makers, and publics, who share core understandings about responsibility” (Mejlgaard et al. 2018a: 761). Thus, the uptake of RRI is concerned with adopting new ways of assessing the societal benefits of technical processes and outputs, but on another and arguably more transformative level, also about promoting culture change amongst individual R&I practitioners, along with enhanced societal engagement and input. While important, such culture change may be uneven and appear as less immediate and tangible than infrastructural and regulatory changes achieved under the OS banner.

Our concern here is that differences in emphasis and prescriptions for change may have significant impacts. Wherever RRI and OS do *not* align, there is a risk that OS’ ascent on the political agenda may displace or counter efforts arising from RRI. It is therefore important to pause and examine the momentum of each direction, and how their seemingly shared focus on ‘opening up’ may distract from very different prescriptions for transforming the R&I system. This concern is especially pressing as RRI will feature less on the EC’s political agenda. Draft plans for Horizon Europe, the upcoming framework programme, do not include plans for a continuation of SwafS or a dedicated work programme with similar aims (Mejlgaard et al. 2018b). A future dominated by OS at the expense of RRI then, may pull attention and resources in different directions.

The differences in prescriptions for ‘opening up’ in OS and RRI become especially clear when examined in relation to topics that are of central importance in both. In the following therefore, we expand on the above accounts of RRI and OS, by comparing their respective emphasis on and articulation of issues key to opening up science to society, in relation to engagement with publics and stakeholders on the one hand, and to interdisciplinary responses to societal challenges, on the other. These two elements are recurring topics of concern within the literature on both RRI and OS, and are thus helpful examples with which to illustrate some of the underlying differences across the two movements.

### Engagement with publics and stakeholders

Publics and stakeholders are invited in to the R&I system in both OS and RRI. Thus far, Open Science can be viewed as a process and ambition oriented towards doability, and thereby the internal processes and structures of doing research and innovation. OS is researcher-driven, with the peer community the most important audience. However, Open Science is also driven by a more outward-looking focus, which may be expressed as an ambition to democratise research. This is visible in an emphasis on the inclusion of “non-institutional participants”, or the public, in the scientific process as “valid producers of knowledge” (European Commission, 2016: 55). Developments in digital technology facilitate the engagement of citizens and society in “completely new ways” ranging from targeted data-gathering activities to contributions of local contextual knowledge (European Commission 2013). Citizen science is viewed by the Commission as having a key role to play in advancing the Commission’s goal of RRI as it “reinforces public engagement and can re-direct research agendas towards issues of concern to citizens” (European Commission, 2016: 54).

Open Science then, appears to share RRI’s focus in ‘opening up’ of all stages of the research process. However, OS’ ambitions are narrower in scope. RRI’s approach to opening up extends to an invitation to publics to co-define the aims and means of technical processes in order to increase their alignment with public values (von Schomberg 2013), while Open Science restricts ambitions for opening up to adjustments and improvements to processes based on quality criteria ultimately rooted in the existing research system. While RRI’s publics are invited to speak back to authoritative experts and immutable facts and are *ostensibly* encouraged to rethink regimes and systemic implications in the broadest sense, OS’ publics operate as citizen scientists collecting or systematising data without necessarily reflecting on or critiquing the broader institutional and societal frameworks for uptake.

In RRI then, lies a view of societal voices and citizens as legitimate conversation partners and beneficiaries of technology and knowledge, while OS can be viewed as articulating a less symmetrical relationship between technical experts and societal voices than that called for in RRI. And while RRI’s prescriptions are motivated by experiences of expert hubris and unintended consequences of emerging technologies, OS’ prescriptions appear dominated more by a sense of opportunity and potential for more and better R&I. The contrast between RRI and OS' approach to engagement with publics and stakeholders is thus one of normative versus pragmatic motivations for opening up R&I to these groups.

### Interdisciplinarity in response to societal challenges

Both RRI and OS aim for societal relevance, and therefore align in different ways with the current emphasis on mission-orientedness in R&I policies and frameworks, which is expected to influence Horizon Europe (Mazzucato 2018). In our view, one could see this claim to relevance as influencing the respective approaches to interdisciplinarity prescribed by each movement. OS advances an agenda of digital research infrastructures that implies a call for a fundamental transformation of existing R&I systems. This agenda draws attention to pragmatic questions of how to construct a functional infrastructure, one in which SSH’s critical capacities and interpretative and exploratory methodologies do not fit; the emphasis here is rather on reducing incommensurability between disciplines and data sets.

In RRI, “interdisciplinarity”, looks quite different than in OS. RRI emphasises explorative methods and the incorporation of value judgments alongside epistemic and mechanistic ones. The aim is to explicitly address concerns around interconnectedness, indeterminacy, and legitimacy with regard to the outputs of R&I processes. Normative justifications are given for the inclusion of non-experts in technical processes. This implies that different SSH fields and societal actors are invited into the research process for different reasons. As a consequence, the explorative and interpretative approaches of humanities and social science traditions are allotted relevance and a clearly articulated function founded in their respective practices or fields of expertise. Thus, interdisciplinarity in RRI fosters processes less marked by the predefined agenda of scientific and technological research communities. While RRI’s focus is a normative one, in which interdisciplinarity is used to probe the desirability of different directions for change, OS’s focus is more pragmatic, pursuing the doable through more and better science. In other words, the envisioned expectations, forms, and degrees of integration of social sciences and humanities (SSH) with technical fields and activities differ in RRI and OS. While we are not familiar with existing empirical examples or studies of the effects and dynamics of SSH in RRI and OS respectively, we suggest that SSH may be seen as facing invitations into two very different regimes for interdisciplinarity, and call for further comparative research on SSH incorporation in RRI and OS.

## Conclusion

This article unpacks and elaborates on the transformative change agendas of RRI and OS with respect to the ambition of opening up to society. That the two movements have contrasting ambitions is suggested by exemplary cases of each movement. In what is arguably an exemplary case of OS’ benefits for example, Fields Medal recipient Timothy Gowers’ *Polymath Project* solved mathematical problems by gathering crowd-sourced contributions from mathematicians all over the world (Cranshaw and Kittur 2011). Here, the benefits of ‘opening up’ can be seen as flowing from scientists opening up their methods of working through innovative use of new technology. By contrast, in RRI, von Schomberg’s (2013) emphasis on the importance of RRI to avoid “blind” market driven innovation, ‘opening up’ can be seen as opening up of professional practices and institutional dynamics in the R&I system to greater civic scrutiny and participation. These two motivations suggest different foci for the work of ‘opening up’, emphasising opportunity and responsibility, respectively. It is pertinent to pause and reflect on such differences now, at a time in which OS appears to be on the rise on the EU political agenda, possibly at the expense of RRI, the momentum of which is slowing.

Our comparison in this article sheds light on what is lost and gained with their changing relative importance, and which of RRI’s contributions risk being overshadowed in forthcoming directions of European research policy. Specifically, we suggest that contrasting prescriptions for ‘opening up’ have implications for key areas of shared focus, as seen in engagement with publics and stakeholders on the one hand, and in interdisciplinarity in response to societal challenges on the other. These contrasts provide a stronger basis for reflecting on the broader implications of the apparent shift in policy attention from RRI to OS on the EU level, and the associated anticipated shift in focus for scholars and scholarship engaged in science-society relations.

The main commonalities between RRI and OS as shown above, is that both operate according to an overarching normative commitment to scientific and technical processes for societal good. In this sense, they both call for transformative institutional, structural, behavioural and methodological changes that ultimately are justified not with reference to science’s innate curiosity or self-contained values, but to areas of application and impact. Notable differences exist however, with respect to the practical recommendations that RRI and OS make. RRI focuses more on producing (ethically and societally) “good” outcomes than on resulting in the (epistemically and functionally) “best” outcomes, while OS for its part remains agnostic about the former and concerns itself almost entirely with the latter, and more often concerns itself with issues of efficiency, optimization, integration and potential.

The distinctions between OS and RRI laid out in this article have implications for us as researchers of ELSA, RRI and related fields. As we strive to stay relevant and make positive contributions to the ways in which science and innovation interact with society, it is important to reflect on the broader implications of the normative programmes we associate with, and to stay attentive to discontinuities in the focus and emphasis of research funders. RRI has been concerned with processes of public participation in science and their impact on the knowledge produced, the notion of justified public trust in science, and the idea of research pursued for the common good. Such topics bring social procedures together with epistemic and ethical considerations and thus raise philosophical challenges. A key philosophical challenge of RRI according to Carrier & Irzik (2019 2), is that all actors work together through inclusive methods to ensure research is done in “interaction with society, for one, and for the benefit of or on behalf of society, for another.” Our comparison however, suggests that the expectations and ambitions for science’s engagement with its publics are changing, and that publics to a lesser degree will be invited to reflect systematically on the structural and long-term implications of research and innovation, under an OS-focused research policy regime. OS’s future efforts, particularly in the area of citizen science, may thus benefit from building on RRI’s achievements in the area of institutionalizing participatory approaches to R&I, rather than abandoning them altogether.

Looking ahead, the contrasts between RRI and OS’ prescriptions for ‘opening up’ are also notable for the relatively higher emphasis on pragmatism and instrumentality in OS. One could speculate that OS’ instrumental focus might allow it to converge more easily with political and institutional goals to attract capital and sustain its momentum as a policy tool than RRI has been able to do so far; the question then becomes, at what cost?

## Data Availability

Not applicable.

## Abbreviations

RRI: Responsible Research and Innovation
OS: Open Science
R&I: Research and innovation
ELSA: Ethical, Legal and Societal Aspects
ICT: Information and communications technology
EC: European Commission
NSF: National Science Foundation
MORRI: Monitoring the Evolution and Benefits of Responsible Research and Innovation
CFCs: Chlorofluorocarbons
LERU: The League of European Research Universities
NFR: Norges forskningråd (The Research Council of Norway)
3Os: Open innovation, open science, open to the world

## Footnotes

1 As observed by Owen and Pansera (2019), researchers in EC-funded projects have variously endeavoured to reconcile this tension between the keys and “more ambitious” understandings of RRI; ranging from attempts to reorient the keys as policy agendas within a broader framing, to pushing the keys to the wayside or focusing only on the keys and not on the broader vision of RRI.

2 The fourth paradigm of scientific research is described as a data-intensive or data-driven science. The first three data paradigms are described as successive historic paradigms starting with the emphasis on 1) empirical findings, 2) testing of theories and 3) computer simulations to understand the world.
